# Supplementary material for: Stochastic search and joint fine-mapping increases accuracy and identifies previously unreported associations in immune-mediated diseases
Source: Nat Commun. 2019 Jul 19;10:3216. doi: 10.1038/s41467-019-11271-0 (PMC6642100; doi:10.1038/s41467-019-11271-0)
Supplement: Supplementary file 1 — Supplementary information [file 41467_2019_11271_MOESM1_ESM.pdf]

Stochastic search and joint fine-mapping increases accuracy and identifies previously unreported associations in immune-mediated diseases

Supplementary Information

Jennifer L Asimit, Daniel B Rainbow, Mary D Fortune, Nastasiya F Grinberg, Linda S Wicker, Chris Wallace

Supplementary Note 1: LD structures corresponding to joint tagging

We consider 3 SNPs. SNPs 1 and 2 are causal and SNP 3 is not causal. The LD correlation matrix between the SNPs is

$$\Sigma = \begin{bmatrix} 1 & r_{12} & r1 \\ r_{12} & 1 & r2 \\ r1 & r2 & 1 \end{bmatrix}$$

Because Σ is a correlation matrix, it must be positive definite, which means r_{12}, r_1, r_2 must satisfy

$$-2 * r_1 * r_2 * r_{12} + r_1^2 + r_2^2 + r_{12}^2 \leq 1 \tag{1}$$

so that r_{12}, r_1, r_2 are constrained to lie within an ellipse. If the true expected Z scores from a joint model against all SNPs is $Z_J = (\zeta_1, \zeta_2, 0)'$, then the expected marginal Z scores are $Z_M \simeq \Sigma Z_J = (z_1, z_2, z_3)'$ where $z_i = E\left(\frac{\beta_i}{\sigma_i}\right)$, and β_i, σ_i are the log odds ratio and its standard error, respectively, for SNP i. The region within the ellipse that correspond to joint tagging is defined by the intersection of

$$z_3 \simeq |\zeta_1 r_1 + \zeta_2 r_2| > |\zeta_1 + \zeta_2 r_{12}| \simeq z_1$$

$$z_3 \simeq |\zeta_1 r_1 + \zeta_2 r_2| > |\zeta_2 + \zeta_1 r_{12}| \simeq z_2$$

There are then 5 unknown parameters which control whether tagging is expected. However, we can make inference under some simplified expectations. For example, if we assume that the two causal SNPs have equal effect sizes measured by Z scores, ie $\zeta_1 = \zeta_2 \Rightarrow z_1 = z_2$, then this reduces to

$$|r_1 + r_2| > |1 + r_{12}|. \tag{2}$$

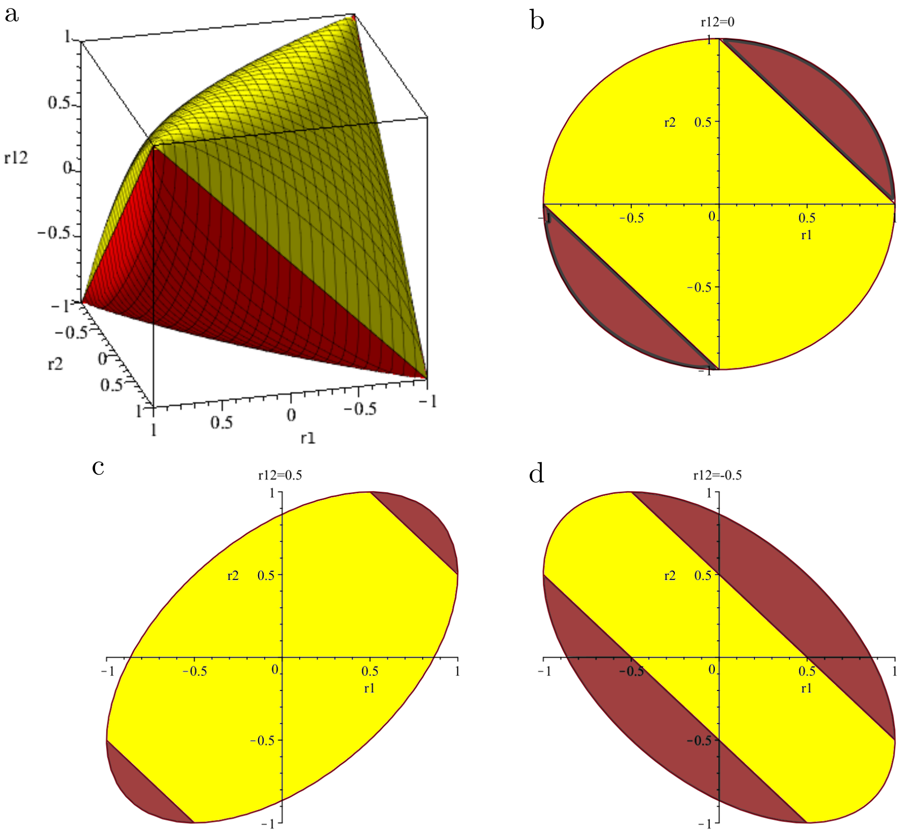

Supplementary Figure 1: **Correlations between 2 causal SNPs and a potential joint tag SNP.** The 3-way correlations between 2 causal SNPs (r_{12}, z axis) and each causal SNP and a potential joint tag (r_1, and r_2, x and y axes respectively) lie in a simplex. **a** Assuming $\zeta_1 = \zeta_2$, then points within this simplex may be coloured according to whether joint tagging is expected (red) or not (yellow) if both causal variants have equal effect sizes. **b**, **c**, **d** show planes through this simplex when the causal variants are uncorrelated ($r_{12} = 0$), positively correlated ($r_{12} = 0.5$) and negatively correlated ($r_{12} = -0.5$) respectively.

Equation (2) thus defines the subset of points within the simplex (within which these 3-way correlations must lie) which correspond to joint tagging under the assumption $\zeta_1 = \zeta_2$. In particular, note that this subset is non-empty - ie joint tagging is possible - even for unlinked causal variants, since setting $r_{12} = 0$ joint tagging requires a solution to the simultaneous inequalities derived from (1), (2):

$$\begin{aligned} |r_1 + r_2| &> 1 \\ r_1^2 + r_2^2 &\leq 1 \end{aligned} \tag{3}$$

and many such solutions exist, e.g. $r_1 = r_2 > 0.5$ (see Supplementary Figure 1).

Alternatively, it may be more realistic to assume that the SNPs have equal odds ratios, β^*. Noting that $\sigma_i^2 \propto f_i(1-f_i)$ where f_i is the MAF of SNP i, we have joint tagging when

$$
\begin{aligned}
z_3 \simeq \left|\frac{\beta^*}{\sqrt{f_1(1-f_1)}}r_1 + \frac{\beta^*}{\sqrt{f_2(1-f_2)}}r_2\right| > \left|\frac{\beta^*}{\sqrt{f_1(1-f_1)}} + \frac{\beta^*}{\sqrt{f_2(1-f_2)}}r_{12}\right| \simeq z_1 \\
z_3 \simeq \left|\frac{\beta^*}{\sqrt{f_1(1-f_1)}}r_1 + \frac{\beta^*}{\sqrt{f_2(1-f_2)}}r_2\right| > \left|\frac{\beta^*}{\sqrt{f_1(1-f_1)}}r_{12} + \frac{\beta^*}{\sqrt{f_2(1-f_2)}}\right| \simeq z_2
\end{aligned}
$$

which reduces to

$$
\begin{aligned}
z_3 \simeq \left|\frac{r_1}{\sqrt{f_1(1-f_1)}} + \frac{r_2}{\sqrt{f_2(1-f_2)}}\right| > \left|\frac{1}{\sqrt{f_1(1-f_1)}} + \frac{r_{12}}{\sqrt{f_2(1-f_2)}}\right| \simeq z_1 \\
z_3 \simeq \left|\frac{r_1}{\sqrt{f_1(1-f_1)}} + \frac{r_2}{\sqrt{f_2(1-f_2)}}\right| > \left|\frac{r_{12}}{\sqrt{f_1(1-f_1)}} + \frac{1}{\sqrt{f_2(1-f_2)}}\right| \simeq z_2
\end{aligned} \tag{4}
$$

Decisions on whether individual observations corresponded to joint tagging in Figure 2b–c were made on the basis of equations (3)-(4).

Supplementary Note 2: Statistical inference of joint versus tag models

In this section, we consider how statistical inference will perform, when comparing joint models to tag models, by evaluating their likelihood ratio and the Bayesian Information Criterion (BIC). Let Z^o be the observed Z scores for the joint 3-SNP model, with $Z^o \sim N(Z_M, \Sigma)$, $Z_M = \Sigma Z_J$.

$$
\begin{aligned}
H_0 : & Z_J = \tilde{z}_J = (0, 0, \tilde{\zeta})' \\
H_1 : & Z_J = z_j = (\zeta_1, \zeta_2, 0)'
\end{aligned}
$$

For simplicity, we assume the MLE of the effect sizes under the appropriate hypothesis are their true values, i.e. $\hat{\zeta}_i = \zeta_i$, $i = 1, 2$, assuming H_1, and $\hat{\tilde{\zeta}} = r_1\zeta_1 + r_2\zeta_2$, assuming H_0. We are interested in evaluating the plausibility of situations in which we would erroneously infer H_0 when H_1 is true. Assuming a likelihood ratio is used for comparison, this would correspond to

$$\frac{\ell(Z_J = (\zeta_1, \zeta_2, 0)'|z_o)}{\ell(Z_J = (0, 0, \tilde{\zeta})'|z_o)} < 1,$$

where the left-hand side is the likelihood ratio of observing Z scores (z_1^o, z_2^o, z_3^o) under H_1 compared to H_0. Since Z^o is Normally distributed, the inequality becomes

$$\frac{(2\pi|\Sigma|)^{-1/2} \exp\left(-\frac{1}{2}(z_o - z_m)'\Sigma^{-1}(z_o - z_m)\right)}{(2\pi|\Sigma|)^{-1/2} \exp\left(-\frac{1}{2}(z_o - \tilde{z}_m)'\Sigma^{-1}(z_o - \tilde{z}_m)\right)} < 1,$$

where $z_m = \Sigma z_J$ and $\tilde{z}_m = \Sigma \tilde{z}_J$. Simplifying, we obtain

$$\begin{aligned} & z_m'\Sigma^{-1}z_m - \tilde{z}_m'\Sigma^{-1}\tilde{z}_m - 2z_o'\Sigma^{-1}(z_m - \tilde{z}_m) > 0 \\ \Rightarrow \qquad & z_J'\Sigma z_J - \tilde{z}_J'\Sigma\tilde{z}_J - 2z_o'(z_J - \tilde{z}_J) > 0, \end{aligned}$$

where we used the fact that Σ is symmetric and expressions for z_m and $\tilde{z}_m$. Substituting z_J, $\tilde{z}_J$ and Σ we obtain

$$2(\tilde{\zeta}z_3^o - \zeta_1 z_1^o - \zeta_2 z_2^o) + \zeta_1^2 + \zeta_2^2 - \tilde{\zeta}^2 + 2\zeta_1\zeta_2 r_{12} > 0. \tag{5}$$

We need to determine whether this condition can be satisfied, and if so, what is the probability of it being satisfied if H_1 is true.

The relative plausibility of the two models can also be assessed using Bayesian information criterion (BIC). Recall that the BIC of a model with k parameters and based on n sample points is $k\ln(n) - 2\hat{\ell}$, where $\hat{\ell}$ is the maximized log-likelihood of the model. Hence, when using BIC, in order for the model under which SNP 3 is causal to be preferred to the model under which SNPs 1 and 2 are causal, the following has to hold

$$\ln(n) - 2\hat{\ell}(Z_J = (0, 0, \tilde{\zeta})'|z_o) < 2\ln(n) - 2\hat{\ell}(Z_J = (\zeta_1, \zeta_2, 0)'|z_o).$$

This gives the following condition

$$2(\tilde{\zeta} z_3^o - \zeta_1 z_1^o - \zeta_2 z_2^o) + \zeta_1^2 + \zeta_2^2 - \tilde{\zeta}^2 + 2\zeta_1\zeta_2 r_{12} > -\ln(n). \tag{6}$$

We now proceed to evaluate probabilities of conditions (5) and (6). We assume $\zeta_1 = \zeta_2 := \zeta$ and $\tilde{\zeta} = \zeta(r_1 + r_2)$. Hence, $Z_J = (\zeta, \zeta, 0)'$ and we have

$$Z^o \sim N\left(\begin{bmatrix} \zeta(1+r_{12}) \\ \zeta(1+r_{12}) \\ \zeta(r_1+r_2) \end{bmatrix}, \Sigma \right),$$

Set

$$W := \tilde{\zeta} z_3^o - \zeta_1 z_1^o - \zeta_2 z_2^o$$

and note that W is Normally distributed and (5) becomes

$$2W - \zeta^2(r_1 + r_2)^2 + 2\zeta^2(1 + r_{12}) > 0, \tag{7}$$

with

$$\mathbb{E}(W) = \zeta^2(r_1 + r_2)^2 - 2\zeta^2(1 + r_{12}) := -\sigma_W^2$$
$$\mathrm{Var}(W) = -\zeta^2(r_1 + r_2)^2 + 2\zeta^2(1 + r_{12}) = \sigma_W^2.$$

Condition $\mathrm{Var}(W) > 0$ evaluates to

$$r_1 + r_2 < \sqrt{2(1 + r_{12})}. \tag{8}$$

We can now evaluate the probability of event (7), $P(\zeta, r_1, r_2, r_{12})$, as

$$P(\zeta, r_1, r_2, r_{12}) = \mathbb{P}(2W + \sigma_W^2 > 0) = 1 - \Phi(1/2\sigma_W)$$
$$= 1 - \Phi\left(\frac{1}{2}|\zeta|\sqrt{2(1 + r_{12}) - (r_1 + r_2)^2}\right).$$

It follows that the probability of choosing H_0 when H_1 is in fact true, is always less than 0.5.

Additionally this probability decreases with the absolute value of effect size ζ and correlation between SNPs 1 and 2, r_{12}, and increases with the squared sum of correlations of causal SNPs 1 and 2 with the tagging SNP 3 (see Supplementary Figure 2).

Similarly, the probability of the BIC condition (6), $P(\zeta, r_1, r_2, r_{12}, n)$, can be calculated to be

$$P(\zeta, r_1, r_2, r_{12}, n) = \mathbb{P}(2W + \sigma_W^2 > -\ln n) = 1 - \Phi\left(\frac{1}{2}\left(\sigma_W - \frac{1}{\sigma_M}\ln(n)\right)\right).$$

Again, the probability of choosing a SNP 3 model, when SNPs 1 and 2 are causal, is non-zero but is no longer bounded above. The general trends remain the same—$P(\zeta, r_1, r_2, r_{12}, n)$ increases with $(r_1+r_2)^2$ and decreases with ζ and r_{12} (although the pool of admissible values (r_1, r_2) increases with r_{12}). Additionally $P(\zeta, r_1, r_2, r_{12}, n)$ slightly increases with the sample size n (see Supplementary Figure 3).

2.1 SNP 3 is causal

If H_0 were in fact true, we would be much less likely to erroneously infer H_1, as shown below. Assume $\zeta_1 = \tilde{\zeta} r_1$ and $\zeta_2 = \tilde{\zeta} r_2$. We have

$$Z^o \sim N\left(\begin{bmatrix} \tilde{\zeta} r_1 \\ \tilde{\zeta} r_1 \\ \tilde{\zeta} \end{bmatrix}, \Sigma\right).$$

Inequality (5) becomes

$$2W - \tilde{\zeta}^2(1 - r_1^2 - r_2^2 - 2r_1r_2r_{12}) > 0, \tag{9}$$

with

$$\mathbb{E}(W) = \tilde{\zeta}^2(1 - r_1^2 - r_2^2)$$
$$\mathrm{Var}(W) = \tilde{\zeta}^2(1 - r_1^2 - r_2^2 + 2r_1r_2r_{12}) = \sigma_W^2.$$

Once again condition $\mathrm{Var}(W) > 0$ yields

$$1 + 2r_1r_2r_{12} > r_1^2 + r_2^2 \tag{10}$$

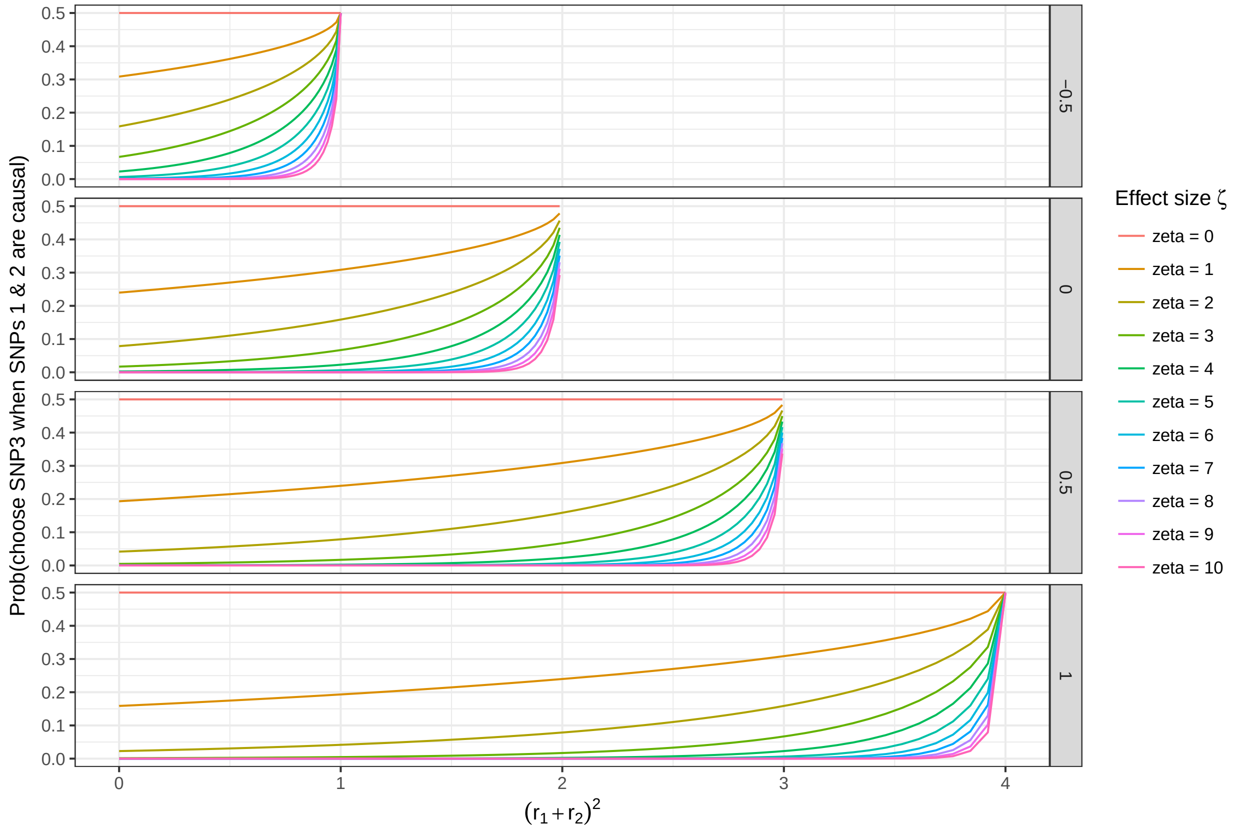

Supplementary Figure 2: **Probability of choosing a SNP 3 model, when SNPs 1 and 2 are causal, under the likelihood ratio setup.** Probability of choosing a model under which SNP 3 is causal, when SNPs 1 and 2 are actually causal, $P(\zeta, r_1, r_2, r_{12})$, under likelihood ratio setup for varying values of $(r_1 + r_2)^2$, squared sum of correlations of SNPs 1 and 2 with SNP 3, different effect sizes ζ, and correlation r_{12} between SNPs 1 and 2 (side panels). Note that there are no combinations of r_1 and r_2 satisfying conditions (1) and (8) when $r_{12} = -1$. $P(\zeta, r_1, r_2, r_{12})$ is bounded above by 0.5 and remains very small for $\zeta > 3$ until $(r_1 + r_2)^2$ is close to its maximum value.

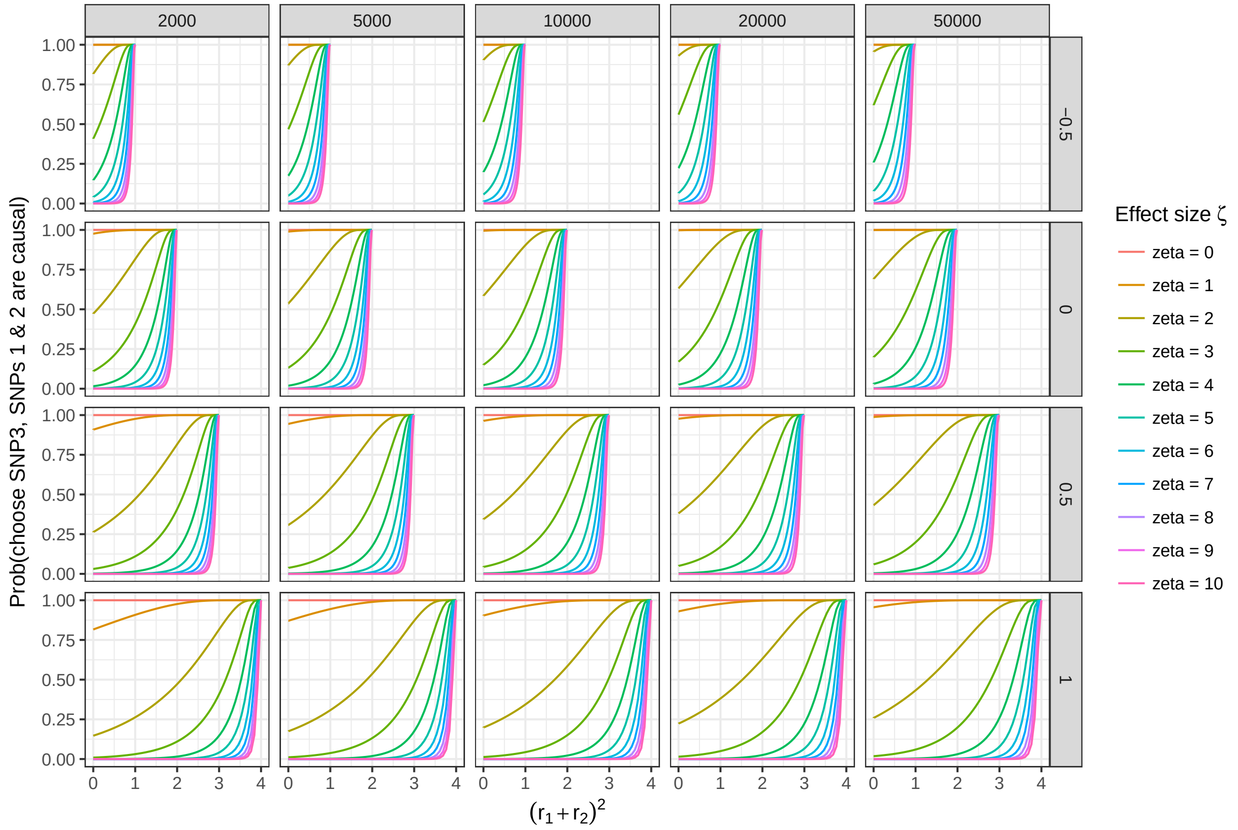

Supplementary Figure 3: **Probability of choosing a SNP 3 model, when SNPs 1 and 2 are causal, under the BIC setup.** Probability of choosing a model under which SNP 3 is causal, when SNPs 1 and 2 are actually causal, $P(\zeta, r_1, r_2, r_{12}, n)$, under BIC setup for varying values of $(r_1 + r_2)^2$, squared sum of correlations of SNPs 1 and 2 with SNP 3, different effect sizes ζ, correlation r_{12} between SNPs 1 and 2 (side panels), and sample size n (top panels). Note that there are no combinations of r_1 and r_2 satisfying conditions (1) and (8) when $r_{12} = -1$. $P(\zeta, r_1, r_2, r_{12}, n)$ remains very small for $\zeta > 3$ until $(r_1 + r_2)^2$ is close to its maximum value.

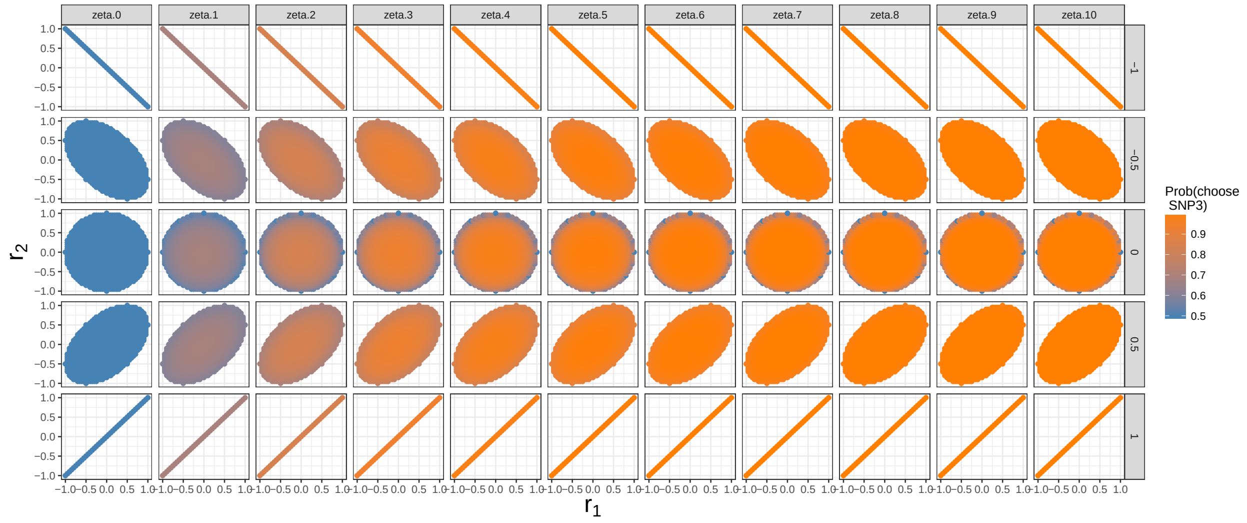

Supplementary Figure 4: **Probability of correctly choosing a SNP 3 model, under a likelihood ratio setup**. Probability of choosing a model under which SNP 3 is actually causal, when SNP 3 is causal, $P(\zeta, r_1, r_2, r_{12})$, under the likelihood ratio setup for varying values of r_1, r_2, different effect sizes ζ and correlation r_{12} between SNPs 1 and 2 (side panels). $P(\zeta, r_1, r_2, r_{12}, n)$ is bounded below by 0.5 and is very close to 1 for all but very extreme values of r_1 and r_2 for effect sizes $\zeta > 4$.

and computing the probability of event (9) we get

$$P(\zeta, r_1, r_2, r_{12}) = 1 - \Phi(-1/2\sigma_W) = 1 - \Phi\left(-\frac{1}{2}|\zeta|\sqrt{1 - r_1^2 - r_2^2 + 2r_1r_2r_{12}}\right).$$

Hence, the probability of picking SNP 3 when it is causal is always greater than 0.5 and increasing with the absolute value of effect size ζ (see Supplementary Figure 4).

Finally we calculate the probability of the BIC condition (6) in a similar fashion

$$P(\zeta, r_1, r_2, r_{12}, n) = 1 - \Phi\left(-\frac{1}{2}\left(\sigma_W + \frac{\ln(n)}{\sigma_W}\right)\right).$$

The probability is non-zero for some combinations of r_1, r_2, r_{12} and ζ, bounded below by 0.5 and increases in sample size n.

Supplementary Note 3: Multinomial Fine-mapping (MFM) Model Description

3.1 The ABF for a multinomial model can be expressed as a function of the ABFs of the dichotomous logistic models

We suppose we observe N individuals, each individual i with response $y_i \in 0, 1, \ldots, m$ for m diseases and a control group, represented by 0. We assume that each individual falls into exactly one class - i.e. that no co-morbid individuals are in the sample - and that each individual has a vector of covariate data $\boldsymbol{x}_i$. A "model" is defined by which elements of $\boldsymbol{x}_i$ are used to fit a regression model to the data, which we write by replacing $\boldsymbol{x}_i$by $\boldsymbol{x}_i^M$ for model M. Let $\phi_{id} = \Pr(y_i = d)$ and $n_d = \sum_i I(y_i = d)$. Then the multinomial regression likelihood is defined by

$$L_M = \prod_{d=0}^{m} \prod_{i:y_i=d} \phi_{id}$$

where ϕ_{id} is estimated from equations

$$\log\left(\frac{\phi_{id}}{\phi_{i0}}\right) = \beta_d' \boldsymbol{x}_i^M, \quad i = 1, \ldots, N, \quad d = 1, \ldots, m$$

and $\sum_{d=0}^{m} \phi_{id} = 1$. Thus,

$$\phi_{id} = \frac{exp(\beta_d' \boldsymbol{x}_i^M)}{1 + \sum_{d=1}^{m} exp(\beta_d' \boldsymbol{x}_i^M)}$$

The corresponding logistic models have likelihoods

$$L_d = \prod_{i:y_i=d} \theta_{id} \times \prod_{i:y_i=0} (1 - \theta_{id})$$

where $\theta_{id} = \Pr(y_i = d | y_i \in \{0, d\})$ and $\log\left(\frac{\theta_{id}}{1-\theta_{id}}\right) = \gamma_d' \boldsymbol{x}_i^M$.

Begg and Gray[1] have shown that $\hat{\gamma}_d = \hat{\beta}_d$, $d \neq 0$, and that

$$\hat{\theta}_{id} = \frac{\hat{\phi}_{id}}{\hat{\phi}_{id} + \hat{\phi}_{i0}}.$$

We wish to show, comparing a specific model to the null model, that the approximate Bayes' factor (ABF) from the multinomial model is approximately proportional to the product of ABF from the logistic models. Accurate approximations to the Bayes' factor comparing the likelihood of the data under model M and the null model (and integrating over the values of the regression coefficients) have been derived [2].

We use an approximation based on the Schwartz inequality which states that, writing B_{M0} for the BF comparing model M with the null model, and with S defined as

$$S = \log \Pr(D|\hat{\theta}_M, M) - \log \Pr(D|\hat{\theta}_0, M_0) - \frac{1}{2}(k_M - k_0)\log(n),$$

$$\frac{S - \log B_{M0}}{\log B_{M0}} \to 0$$

as sample size $n \to \infty$, where k_j denotes the length of the parameter vector θ_j whose maximum likelihood estimate is denoted $\hat{\theta}_j$. Thus, S can be used as an ABF.

Recall we use the term "configuration" to describe a set of m models, $M_1, M_2, \ldots M_m$ such that M_k is the model used to describe case group k. Under the multinomial model, the log(ABF) can be written

$$\begin{aligned} B^M = &\sum_{d=1}^{m} \sum_{i:y_i=d} \hat{\beta}_d' \boldsymbol{x}_i - \sum_i \log\left(1 + \sum_{d=1}^{m} \exp(\hat{\beta}_d' \boldsymbol{x}_i)\right) \\ &- \sum_{d=1}^{m} \sum_{i:y_i=d} \hat{\beta}_{0d} + \sum_i \log\left(1 + \sum_{d=1}^{m} \exp(\hat{\beta}_{0d})\right) \\ &- \frac{1}{2}(k_C - m)\log(N) \end{aligned}$$

where $k_C = \sum_d k_d$ is the total number of parameters in component models $M_1, \ldots, M_m$, and $\hat{\beta}_d$ are the MLE of β under model M_d relating to disease d and $\hat{\beta}_{0d}$ are the MLE of β under the null

(intercept only) model. Under the logistic model for disease d, the log(ABF) can be written

$$\begin{aligned} log(B_d^L) = &\sum_{i:y_i=d} \hat{\beta}_d' \boldsymbol{x}_i - \sum_{i:y_i \in \{0,d\}} \log(1+e^{\hat{\beta}_d' \boldsymbol{x}_i}) \\ &- \sum_{i:y_i=d} \hat{\beta}_{0d} - \sum_{i:y_i \in \{0,d\}} \log(1+e^{\hat{\beta}_{0d}}) \\ &- \frac{1}{2}(k_d - 1)\log(n_d + n_0) \end{aligned}$$

So that the difference between B^M and $\sum_d B_d^L$ is

$$\begin{aligned} D = &\sum_i \left[\log\left(1 + \sum_{d=1}^m \exp(\hat{\beta}_{0d})\right) - \log\left(1 + \sum_{d=1}^m \exp(\hat{\beta}_d' \boldsymbol{x}_i)\right)\right] \\ &+ \sum_{d=1}^m \sum_{i:y_i \in \{0,d\}} \left[\log\left(1 + e^{\hat{\beta}_d' \boldsymbol{x}_i}\right) - \log\left(1 + e^{\hat{\beta}_{0d}}\right)\right] \\ &+ \frac{1}{2}\sum_d (k_d - 1)\log(n_d + n_0) - \frac{1}{2}\log(N)(k_C - m) \end{aligned}$$

Set

$$\begin{aligned} \eta &= \frac{1}{2}\sum_d (k_d - 1)\log(n_d + n_0) - \frac{1}{2}\log(N)\sum_d (k_d - 1) \\ &= \frac{1}{2}\sum_d (k_d - 1) log\left(\frac{n_d + n_0}{N}\right) \end{aligned} \tag{11}$$

We show next that $D - \eta \simeq 0$.

Recall $\phi_{id} = Pr(y_i = d)$, and note that under model M, $\log \hat{\phi}_{i0} = -log\left(1 + \sum_{d=1}^m \exp(\hat{\beta}_d' \boldsymbol{x}_i)\right)$. Note also that $\hat{\beta}_{0d} = \log(n_d/n_0)$ so that

$$\begin{aligned} -\log\left(1 + \sum_{d=1}^m \exp(\hat{\beta}_{0d})\right) &= \log(n_0/N), \quad \text{and} \\ -\log\left(1 + exp(\hat{\beta}_{0d})\right) &= \log\left(n_0/(n_0 + n_d)\right). \end{aligned}$$

Thus

$$D - \eta = -N \log(n_0/N) + \sum_i \log \hat{\phi}_{i0} - \sum_{d=1}^{m} \sum_{i:y_i \in \{0,d\}} \log \frac{\hat{\phi}_{i0}}{\hat{\phi}_{i0} + \hat{\phi}_{id}} + \sum_{d=1}^{m} (n_0 + n_d) \log \frac{n_0}{n_0 + n_d}$$

Now, note that $\frac{1}{N} \sum_i \hat{\phi}_{i0} = n_0/N$. We consider $\sum_i \log(\hat{\phi}_{i0})$ as a sum of Taylor series expansions of $\log \hat{\phi}_{i0}$ about $\frac{n_0}{N}$:

$$\begin{aligned}
\sum_i \log \hat{\phi}_{i0} &\simeq \sum_i \left(\log \left(\frac{n_0}{N} \right) + \frac{N}{n_0} \right) \left(\hat{\phi}_{i0} - \frac{n_0}{N} \right) + O \left(\left(\hat{\phi}_{i0} - \frac{n_0}{N} \right)^2 \right) \\
&\simeq N \log \left(\frac{n_0}{N} \right) + \frac{N}{n_0} \sum_i \left(\hat{\phi}_{i0} - \frac{n_0}{N} \right) \\
&= N \log \left(\frac{n_0}{N} \right) + \frac{N}{n_0} (n_0 - n_0) \\
&= N \log \left(\frac{n_0}{N} \right)
\end{aligned}$$

neglecting terms in $O\left(\left(\hat{\phi}_{i0} - \frac{n_0}{N} \right)^2 \right)$ and smaller.

Similarly,

$$\sum_{i:y_i \in \{0,d\}} \log \frac{\hat{\phi}_{i0}}{\hat{\phi}_{i0} + \hat{\phi}_{id}} = \sum_{i:y_i \in \{0,d\}} \log \hat{\theta}_{i0} \ \simeq \ (n_0 + n_d) \log \left(\frac{n_0}{n_0 + n_d} \right)$$

Therefore,

$$\begin{aligned}
D - \eta &\simeq -N \log(n_0/N) + N \log(n_0/N) - \sum_{d=1}^{m} (n_0 + n_d) \log \left(\frac{n_0}{n_0 + n_d} \right) + \sum_{d=1}^{m} (n_0 + n_d) \log \left(\frac{n_0}{n_0 + n_d} \right) \\
&= 0
\end{aligned}$$

so that

$$B^M \simeq \eta + \sum_d B_d^L \tag{12}$$

To confirm the accuracy of this approximation defined by equations (11)– (12), we simulated genetic data for varying numbers of cases (two diseases) and controls and calculated logistic ABFs for all possible models using the R package BMA. From these, we calculated the summed log

ABF for all possible configurations. For comparison we calculated the log ABF for the comparitor multinomial model directly using the R package mlogitBMA. Finally, we regressed the multinomial log ABF on the summed logistic log ABFs and stored the estimated intercept slope and R^2 of this final linear regresion model. For each dataset, we also calculated the univariate p values for each SNP and disease, and stored the minimum p value for each disease. We repeated this procedure 15,000 times, and found that when the minimum p value was below 10^{-7} that the multinomial and summed logistic log ABFs were linearly related ($R^2 \simeq 1$) with a slope of 1, indicating the the multinomial ABF could be expressed as approximately proportional to the product of the logistic ABFs (Supplementary Figure 5). For larger p values, the approximation was less exact, with the average slope of the regression approaching 1.02 for datasets with minimum p values around 0.01, but R^2 remaining very high, at > 0.9996 for all simulations. Given that the accepted threshold for genomewide significance is typically $< 10^{-8}$, we concluded that this approximation was valid in the range of datasets for which fine mapping might be useful.

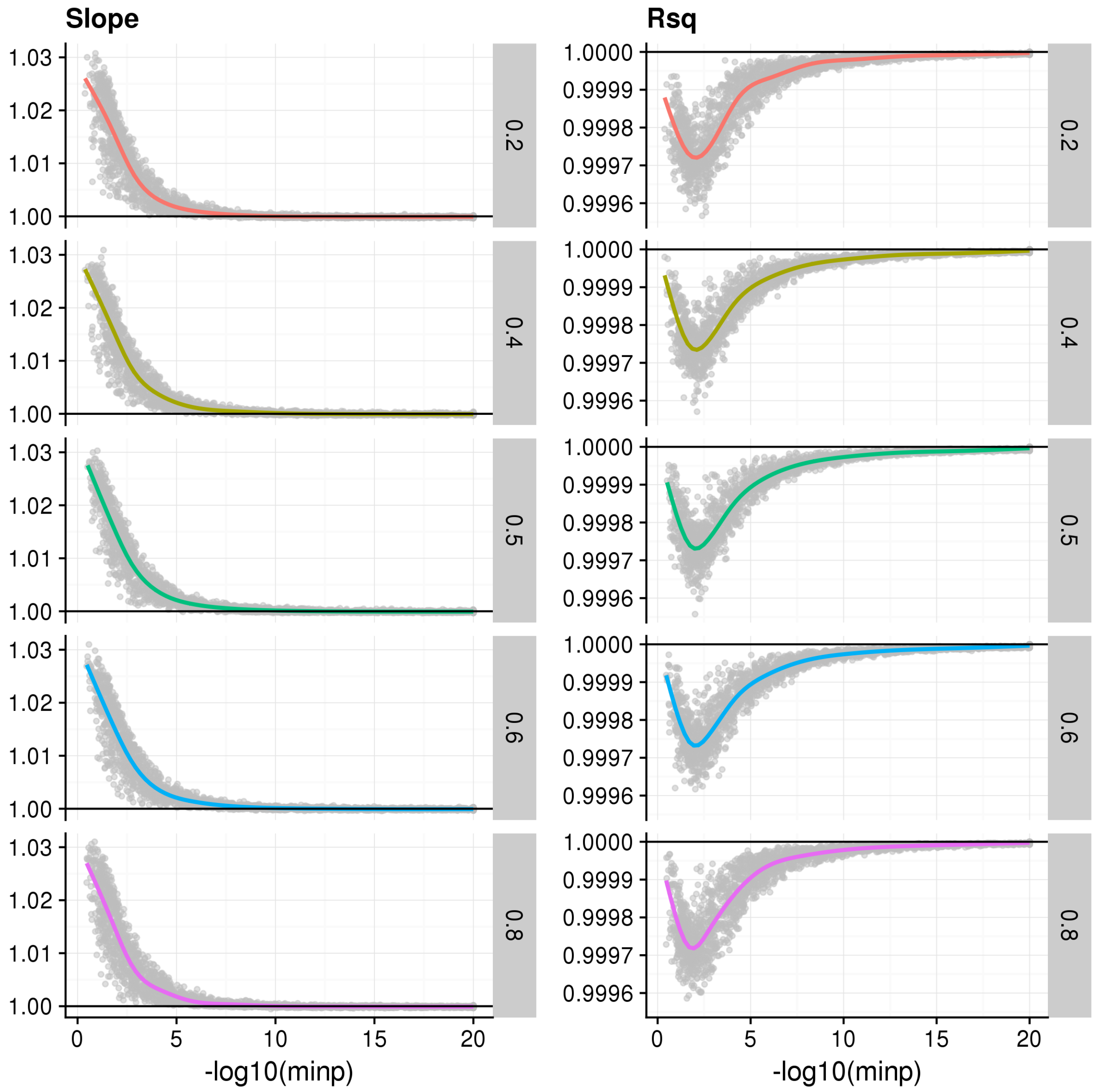

Supplementary Figure 5: **Comparison of log ABF for a multinomial model with the sum of log ABF for component logistic models.** We simulated case-control and genetic data for varying sample sizes, effect sizes, and number of causal variants. We regressed the multinomial log ABF on the sum of logistic log ABFs and found the approximation was valid ($R^2 > 0.9996$ and slope$\simeq 1$) when the minimum p value in both datasets (x-axis) was $< 10^{-8}$. Points represent the individual estimates of slope (left column) and R^2 (right column). Rows are stratified according to the proportion of cases of disease 1 in the simulated sample.

3.2 Memory-efficient calculation of the marginal posteriors for each disease

Let us consider possible models M_i, $i = 1, \ldots, n$. Each model i has a prior p_i, and a Bayes factor for disease 1 and 2, b_i and d_i respectively under a logistic model with k_i parameters. We show above that the joint approximate Bayes factor for a configuration

$$C_{i,j} = M_i \text{ for disease } 1, M_j \text{ for disease } 2$$

is a function of the ABFs from the dichotomous logistic models

$$B_{ij} \propto b_i' d_j',$$

where

$$b_i' = b_i \times \exp(\eta_1(M_i)),$$

$$d_j' = d_j \times \exp(\eta_2(M_j))$$

and

$$\eta_l(M_i) = \exp\left((k_i - 1) \times \frac{1}{2} \log\left(\frac{n_l + n_0}{N}\right)\right), \quad l = 1, 2$$

Thus, the posterior for configuration $C_{i,j}$ is

$$PP_{ij} \propto Pr(C_{i,j}) b_i' d_j'.$$

We incorporate our prior belief on shared causal variants between diseases by upweighting configurations corresponding to such sharing compared to those that don't. We set

$$Pr(C_{i,j}) = p_i p_j \kappa^{M_i \cap M_j \neq \varnothing} \tau_{ij}$$

κ is the upweighting factor, and τ_{ij} is a normalisation factor, chosen to ensure that

$$\sum_{i:|M_i|=m,j:|M_j|=l} p_i p_j = \sum_{i:|M_i|=m,j:|M_j|=l} p_i p_j \kappa^{M_i \cap M_j \neq \varnothing} \tau_{ij} \tag{13}$$

ie, that the prior belief for a configuration corresponding to given model sizes doesn't vary with κ. The equality in (13) implies

$$\binom{n}{m}\binom{n}{l}\pi(m)\pi(l) = \tau_{ij}\pi(m)\pi(l)\left[\binom{n}{m}\binom{n-m}{l} + \kappa\binom{n}{m}\left(\binom{n}{l} - \binom{n-m}{l}\right)\right]$$

$$\tau_{ij} = \frac{\binom{n}{l}}{\binom{n-m}{l} + \kappa\left[\binom{n}{l} - \binom{n-m}{l}\right]} \tag{14}$$

for models M_i and M_j with sizes m and l respectively.

Considering the form of the marginal model posterior probabilities for each disease helps understand how $\kappa > 1$ allows information from disease 2 to be used in our inference for disease 1.

The posterior probability of M_1 for disease 1 is proportional to a sum of the posterior probabilities of all configurations $C_{1,j}$, $j = 1, \ldots, n$. Let $I_{i,j}$ be an indicator function, taking the value 1 if $M_i \cap M_j \neq \varnothing$ and 0 otherwise. Then

$$\begin{aligned}
Pr(M_i \text{ for disease 1}|\text{Data}) &\propto \sum_j p_i p_j b'_i d'_j \times \kappa^{I_{i,j}} \tau_{ij} \\
&= p_i b'_i \left(\sum_{j:I_{i,j}=0} \tau_{ij} p_j d'_j + \kappa \sum_{j:I_{i,j}=i} \tau_{ij} p_j d'_j \right) \\
&= p_i b'_i \left(\sum_j \tau_{ij} p_j d'_j + (\kappa - 1) \sum_{j:I_{i,j}=i} \tau_{ij} p_j d'_j \right) \\
&= p_i b'_i \left(1 + (\kappa - 1) \frac{\sum_{j:I_{i,j}=1} \tau_{ij} p_j d'_j}{\sum_j \tau_{ij} p_j d'_j} \right)
\end{aligned}$$

Noting the similarity to

$$Pr(M_i \text{ for disease 1}|\text{Data for disease 1 only}) \propto b_i p_i$$

we can see that information from disease 2 enters by modifying the prior for model 1 according the posterior support for disease 2 for models that contain any overlap with M_1.

With $n > 2$ diseases, each disease may share causal variants with $n-1$ other diseases. We have more choices now, in terms of how to formulate the joint model - do we upweight further configurations that display sharing between more than 2 diseases? Given the interpretation above of the marginal posterior for one disease, we chose to focus on pairwise sharing of each of $n-1$ other diseases with a single disease of interest. This implies that, if our focus is on disease 1, we consider a prior of the form

$$\pi(C_{ijk}) \propto p_i p_j p_k \kappa^{I(M_i \cap M_j \neq \varnothing)} \kappa^{I(M_i \cap M_k \neq \varnothing)} \tau_{ij} \tau_{ik}$$

(and similar for four or more diseases). This corresponds to a marginal posterior for disease 1 (whose models are indexed by i)

$$Pr(M_i \text{ for disease 1}|\text{Data}) \propto p_i b'_i \left(1 + (\kappa - 1)\frac{\sum_{j:I_{i,j}=1} \tau_{ij} p_j d'_j}{\sum_j \tau_{ij} p_j d'_j}\right) \left(1 + (\kappa - 1)\frac{\sum_{k:I_{i,k}=1} \tau_{ik} p_k d'_k}{\sum_k \tau_{ik} p_k d'_k}\right)$$

This formulation also enables memory efficient calculation of the individual disease marginal posteriors, by stepping through the sums over all configurations, storing only the contents of each large bracket on the right hand side.

As before, so that the prior on any given model size is independent of κ, we have

$$\sum_{i:|M_i|=m, j:|M_j|=l, k:|M_k|=o} p_i p_j p_k = \sum_{i:|M_i|=m, j:|M_j|=l, k:|M_k|=o} p_i p_j p_k \kappa^{M_i \cap M_j \neq \varnothing} \tau_{ij} \kappa^{M_i \cap M_k \neq \varnothing} \tau_{ik}$$

which leads to

$$\begin{aligned} \tau_{ij}\tau_{ik} &= \frac{\binom{n}{o}\binom{n}{l}}{\binom{n-m}{o}\binom{n-m}{l} + \kappa\left[\left(\binom{n}{l} - \binom{n-m}{l}\right)\binom{n-m}{o} + \left(\binom{n}{o} - \binom{n-m}{o}\right)\binom{n-m}{l}\right] + \kappa^2\left(\binom{n}{o} - \binom{n-m}{o}\right)\left(\binom{n}{l} - \binom{n-m}{l}\right)} \\ &= \frac{\binom{n}{l}\binom{n}{o}}{\left(\kappa\binom{n}{l} - \kappa\binom{n-m}{l} + \binom{n-m}{l}\right)\left(\kappa\binom{n}{o} - \kappa\binom{n-m}{o} + \binom{n-m}{o}\right)} \end{aligned}$$

for models M_i, M_j, M_k with sizes m, l, o respectively, which is solved for τ_{ij}, τ_{ik} as given by

equation (14).

3.3 Choice of κ

It may be hard to directly elicit values for the prior parameter κ, that upweights configurations with pairwise sharing of variants between diseases vs configurations without sharing. We set out here how a value for κ may be derived from a quantity that may be more easily elicited - the probability that a pair of diseases share any causal variant (with either concordant or discordant direction of effect) within a region that they both show association, which we denote P_κ.

Recall the prior for a configuration specified by model M_i for disease 1 and model M_j for disease 2 is

$$\Pr(C_{i,j}) \propto p_i p_j \kappa^{I(M_i \cap M_j \neq \varnothing)} \tau_{ij}.$$

where

$$\tau_{ij} = \frac{\binom{n}{n_j}}{\left[\binom{n}{n_j} - \binom{n-n_i}{n_j}\right]\kappa + \binom{n-n_i}{n_j}} = \frac{\binom{n}{n_i}}{\left[\binom{n}{n_i} - \binom{n-n_j}{n_i}\right]\kappa + \binom{n-n_j}{n_i}}$$

Note that, given n SNPs in a region, and using $n_i,\ n_j$ to denote the sizes of models $M_i,\ M_j$ respectively, then the number of models that can be selected with size n_i is $\binom{n}{n_i}$, the number of configurations with model sizes $n_i,\ n_j$ is $\binom{n}{n_i}\binom{n}{n_j}$, and the number of these that contain no shared causal variants is $\binom{n}{n_i}[\binom{n-n_i}{n_j}$ (equivalently, the number which contain at least one shared causal variant is $\binom{n}{n_i}[\binom{n}{n_j} - \binom{n-n_i}{n_j}]$). The prior probability of no sharing in causal variant models is

$$\begin{aligned} P_0 &= \sum_m \sum_l \binom{n}{m}\binom{n-m}{l} \times \frac{\pi(m)}{\binom{n}{m}} \frac{\pi(l)}{\binom{n}{l}} \times \frac{\binom{n}{l}}{[\binom{n}{l} - \binom{n-m}{l}]\,\kappa + \binom{n-m}{l}} \\ &= \sum_m \sum_l \frac{\pi(m)\pi(l)}{\left[\binom{n}{l}/\binom{n-l}{m} - 1\right]\kappa + 1} \end{aligned}$$

Then, assuming our prior probability that two diseases share no causal variants in the region of interest is F_0, κ may be found by numerically solving the equation

$$\frac{F_0}{1-F_0} = \frac{P_0}{1-P_0}$$

which can be set to any elicited value, and numerically solved for κ.

For $d > 2$ diseases, P_0 becomes

$$P_0(d) = \sum_m \pi(m) \left(\sum_l \binom{n-m}{l} \frac{\pi(l)}{\left[\binom{n}{l} - \binom{n-m}{l}\right] \kappa + \binom{n-m}{l}} \right)^{d-1}$$

but we have to be careful about specifying the prior probability for no pairwise sharing. If the other diseases were totally independent, a natural prior value would be F_0^{d-1}. If the other diseases were totally dependent, then the prior would remain at F_0. In the absence of strong prior knowledge about this, we suggest that $F_0^{\sqrt{d-1}}$ is a sensible compromise, but that both extreme values F_0 and F_0^{d-1} should also be explored.

References

[1] Begg, C. B. and Gray, R. Calculation of polychotomous logistic regression parameters using individualized regressions. *Biometrika* **71**(1), 11–18 (1984).

[2] Raftery, A. E. Approximate Bayes factors and accounting for model uncertainty in generalised linear models. *Biometrika* **83**(2), 251–266 (1996).

Supplementary Figures

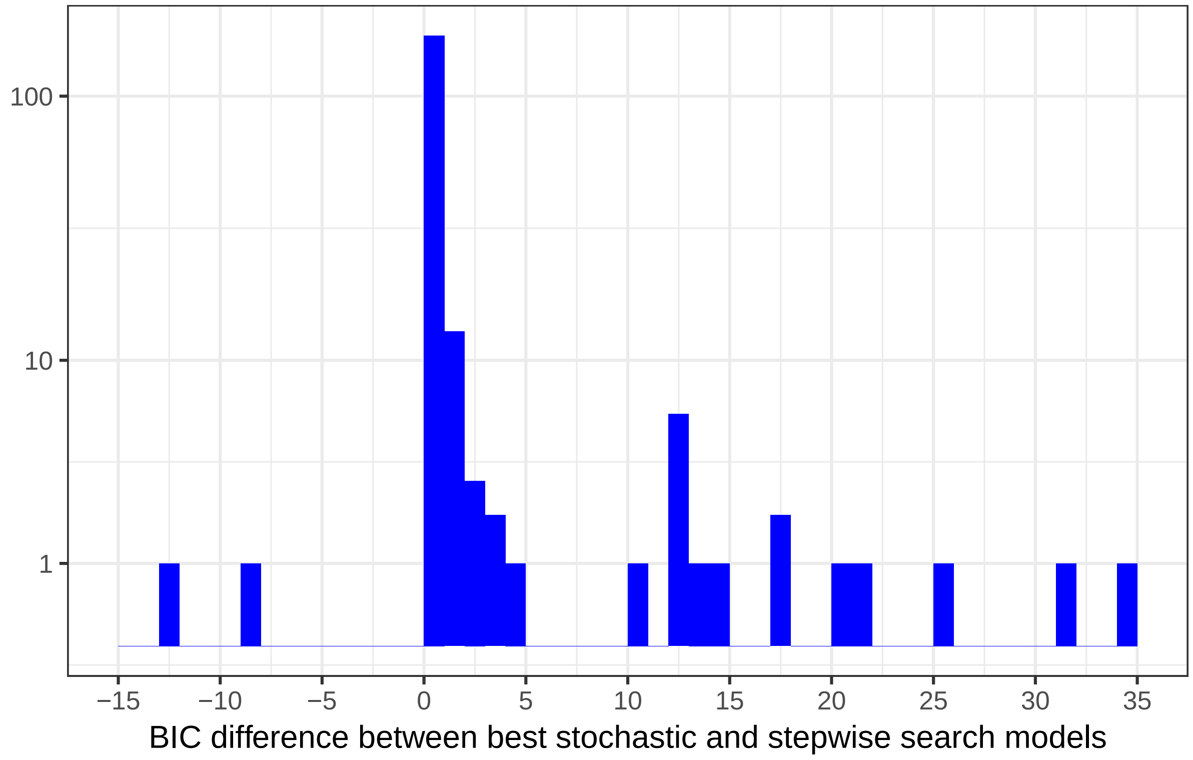

Supplementary Figure 6: **Histogram of BIC(Stepwise Search) - BIC(Stochastic Search) for the best stochastic and stepwise search models from each region-disease analysis.** Stochastic search tends to have a BIC that is the same or smaller as that from stepwise search, indicating a fit that is at least as good or better than stepwise search.The BIC is calculated using the best SNP model from stochastic search, and for two regions, the null model is preferred over each individual SNP model, coinciding with the two instances of stepwise appearing to select a better-fitting model than stochastic search. However, in these two instances, the best non-null model selected by stochastic search agrees with that of stepwise search.

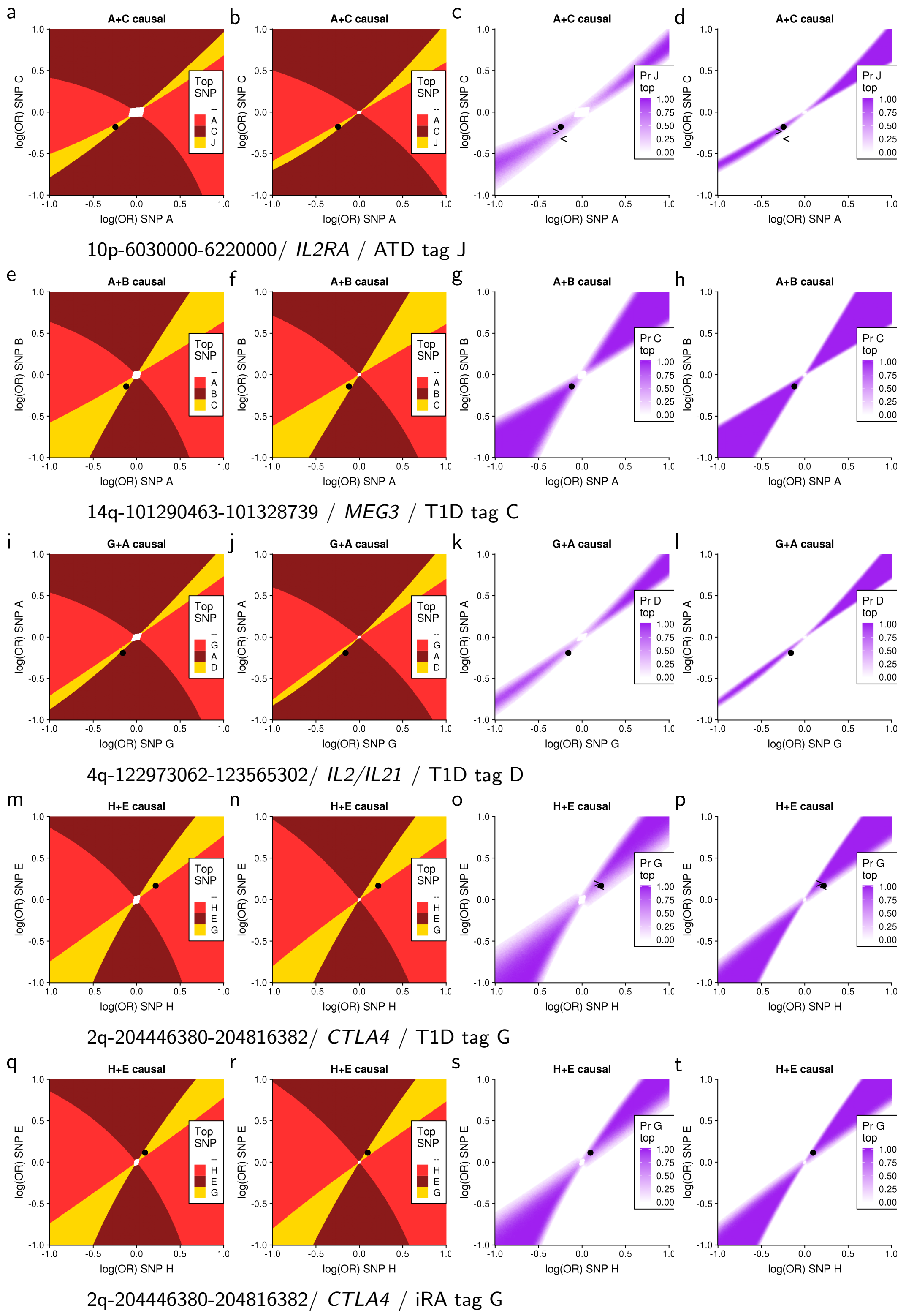

Supplementary Figure 7: **Stepwise regression behaviour when there are two causal SNPs.** Each plot **a-t** is a 2x2 grid, where each point represents a single two SNP causal model, specified by effects (log odds ratio) for the causal SNPs on the X and Y axes. First two columns: "Sunbeam plots" show which SNP is expected to be selected first in a step wise search at each position. Last two columns: "Probability plots" show the probability that the tag SNP was first selected in a stepwise search. Columns 1, 3: Sample sizes from relevant disease dataset; Column 2,4: sample size of 50000 cases and 50000 controls. Two sample sizes are shown so the dependence on small sample size can be evaluated. The black dot shows where the observed disease data lie.

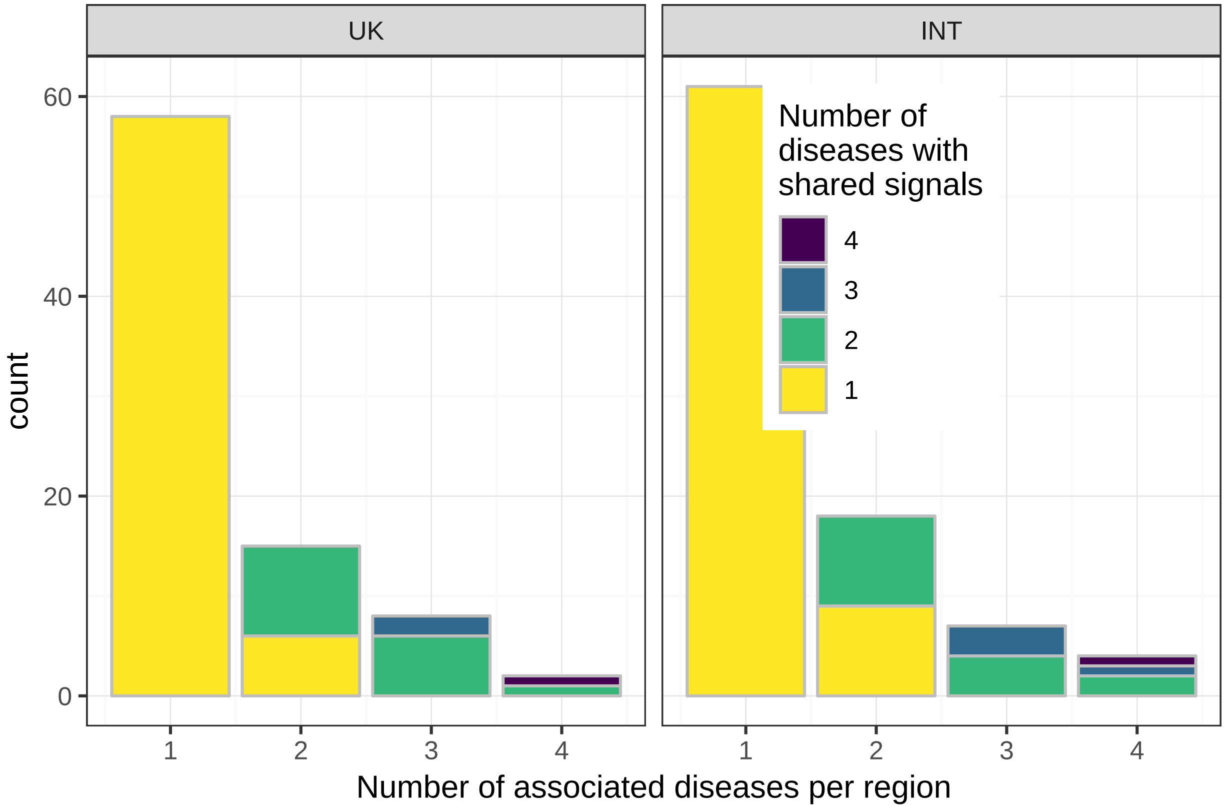

Supplementary Figure 8: **Distribution of the number of associated diseases per region.** Frequency of the number of associated diseases per region, partitioned by whether signals are shared between diseases, for UK samples (left) and international samples (right). We consider a signal to be shared when there exists a SNP group with MPPI > 0.5 for more than one disease.

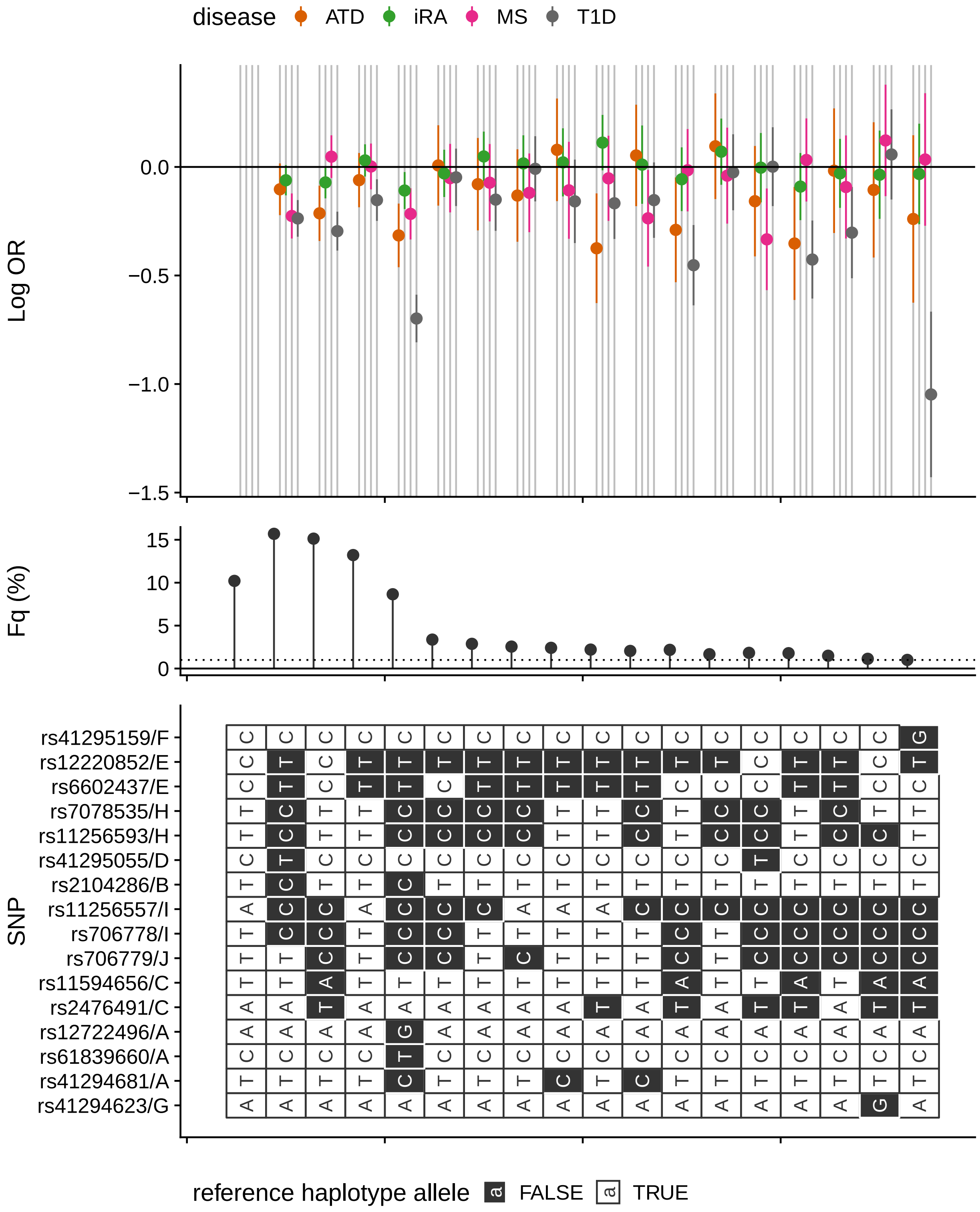

Supplementary Figure 9: **Haplotype analysis of SNPs selected by stepwise search and GUESSFM for IMD in region 10p-6030000-6220000.** Representative SNPs from each SNP group is shown. Each row represents one SNP, with possible alleles colour coded according to major or minor. Each column is a haplotype - a specific combination of alleles across all SNPs - with frequency in UK controls and effect on disease risk (log OR + 95CI). MAF is shown as a percentage on a log scale to allow frequencies of rarer haplotypes to be distinguished.

Supplementary Tables

Country\ Phenotype	Autoimmune Thyroid Disease (ATD)	Celiac Disease (CEL/iCEL)	Juvenile Idiopathic Arthritis (JIA)	Multiple Sclerosis (MS)	Rheumatoid Arthritis (RA/iRA)	Type 1 Diabetes (T1D)	CONTROL
Spain (ES)	0	0	0	0	807	0	399
IndiaPunjab	0	229	0	0	0	0	391
Italy	0	1374	0	0	0	0	1255
Netherlands	0	1104	0	0	648	0	2007
Poland	0	505	0	0	0	0	533
Southeastern Europe (SEE)	0	0	0	0	2762	0	1940
Southern Europe (SEU)	0	0	0	0	852	0	963
Spain-CEGEG	0	545	0	0	0	0	308
SpainMadrid	0	556	0	0	0	0	320
**UK**	**2772**	**7728**	**1214**	**4461**	**3870**	**6681**	**12747**
US	0	0	0	0	2536	0	2134
**Total**	**2772**	**12041**	**1214**	**4461**	**11475**	**6681**	**22997**

Supplementary Table 1 : Immune-mediated disease data sample sizes by country and phenotype. We ran analyses on UK-only (ATD, CEL, JIA, MS, RA, T1D) and international (all countries, iCEL, iRA) samples.

Region	Disease	Stepwise	Stochastic GPP	Stochastic	Stochastic GPP
1q-172650685-172940450	CEL	A	0.0939	A+C	0.796
	iCEL	A	5.82E-05	A+D	0.858
1q-206802440-207032751	T1D	C	0.207	C+B	0.511
2q-191873553-192007734	iRA	G	0.1	G+C	0.521
2q-204446380-204816382	iCEL	I	0.14	I+K	0.351
3p-45929800-46650993	iCEL	A+G	0.0544	A+B+G	0.598
3q-159586299-159754507	iCEL	A+D	0.0766	A+D+E	0.863
4q-122973062-123565302	iCEL	G	0.141	G+E	0.729
6q-127952182-128340790	iCEL	C	0.105	C+A	0.705
6q-137882875-138275085	iCEL	A+C	0.376	A+C+H	0.402
	iRA	C	0.48	C+F	0.484
6q-159322326-159541830	CEL	C	0.348	C+D	0.457
10p-6030000-6220000	T1D	A+C+E	1.33E-08	A+C+E+F	0.622
13q-100036418-100108807	iCEL	B	0.273	B+D	0.646
14q-69168821-69318062	JIA	D	0.148	C+D	0.767
18p-12738413-12924117	T1D	C	0.0175	C+E	0.895
19p-10396336-10628468	T1D	C	0.0085	A+C	0.892
21q-43810084-43887145	T1D	C	0.0364	C+D	0.767

Supplementary Table 2: Region-disease combinations where the best stepwise model is nested within the best stochastic search model. The best stepwise model is listed according to the SNP group(s) that the SNP(s) belong to. Highest group posterior probability (GPP) was used to select the best model for stochastic search and the stochastic GPP is also given for the stepwise model.

(a) Stochastic Search, J=0.8

Model/N	1000	2000	3000	4000	5000
A	0.005	2.11E-03	4.09E-04	4.18E-06	0
C	0.015	0.051	0.017	1.46E-03	1.31E-04
J	0.173	0.686	0.950	0.990	0.996
A+C	1.19E-04	7.59E-05	4.64E-04	6.62E-04	0
A+C+J	0	0	0	0	0
null	0.756	0.244	0.022	1.42E-03	0
other	0.051	0.017	9.75E-03	6.29E-03	3.92E-03

(b) Step-wise Regression, J=0.8

Model/N	1000	2000	3000	4000	5000
A	0	0	0	0	0
C	0	0	0.01	0	0
J	0.01	0.46	0.91	0.99	1
A+C	0	0	0	0	0
A+C+J	0	0	0	0	0
null	0.98	0.54	0.08	0.01	0
other	0.01	0	0	0	0

Supplementary Table 3: Case-control simulations with one causal variant, J, in *IL2RA*.
(a) Model mean posterior probability (GUESSFM; stochastic search) and **(b)** Mean model selection probability (stepwise regression) for simulated data having causal variant J with OR=0.8. Case-control data were simulated with the characteristics of the *IL2RA* region, and there were 100 replications. Sample sizes were *N* cases, *N* controls for *N*=1000 to 5000 and are listed by column.

a) Stochastic Search, A=0.81,C=0.74

Model/N	1000	2000	3000	4000	5000
Null	0.252	0	0	0	0
A+C	0.049	0.175	0.440	0.539	0.661
C	0.490	0.701	0.510	0.430	0.258
J	0.106	0.081	0.010	7.57E-03	0.011
other	0.102	0.042	0.040	0.024	0.071

(b) Stepwise Regression, A=0.81,C=0.74

Model/N	1000	2000	3000	4000	5000
null	0.86	0.21	0.02	0	0
A+C	0	0	0.02	0.04	0.18
C	0.1	0.68	0.89	0.9	0.76
J	0.04	0.1	0.07	0.06	0.06
other	0	0.01	0	0	0

c) Stochastic Search, A=0.74,C=0.81

Model/N	1000	2000	3000	4000	5000
null	0.523	0.115	0.020	3.25E-06	0
A+C	0.056	0.363	0.550	0.868	0.898
C	0.125	0.179	0.203	0.056	1.16E-02
J	0.150	0.259	0.106	0.052	0.024
other	0.147	8.37E-02	0.120	2.43E-02	6.65E-02

d) Stepwise Regression, A=0.74,C=0.81

Model/N	1000	2000	3000	4000	5000
null	0.99	0.77	0.45	0.18	0.04
A	0	0.02	0	0.07	0.01
A+C	0	0	0	0.17	0.19
C	0	0.05	0.21	0.3	0.19
J	0.01	0.16	0.28	0.26	0.55
other	0	0	0.06	0.02	0.02

Supplementary Table 4: Case-control simulations with two causal variant, A+C, in *IL2RA*. (a,c) Model mean posterior probability (GUESSFM; stochastic search) and **(b,d)** Mean model selection probability (stepwise regression) for simulated data having causal variants A + C, odds ratios A:0.81, C:0.74 **(a,b)**, A:0.74, C:0.81 **(c,d)**. Case-control data were simulated with the characteristics of the *IL2RA* region, and there were 100 replications. Sample sizes were *N* cases, *N* controls for *N*=1000 to 5000 and are listed by column.

a) Stochastic Search, G=1.25

Model/N	1000	2000	3000	4000	5000
G	0.329	0.824	0.918	0.959	0.988
H	0.094	0.093	0.058	0.020	1.37E-03
null	0.553	0.067	2.34E-03	3.55E-03	1.14E-06

(b) Stepwise Regression, G=1.25

Model/N	1000	2000	3000	4000	5000
G	0.06	0.64	0.93	0.97	1
null	0.94	0.33	0	0	0

Supplementary Table 5: Case-control simulations with one causal variant, G, in *CTLA4*.
(a) Model mean posterior probability (GUESSFM; stochastic search) and **(b)** Mean model selection probability (stepwise regression) for simulated data having causal variant G with OR=1.25. Case-control data were simulated with the characteristics of the *CTLA4* region, and there were 100 replications. Sample sizes were *N* cases, *N* controls for *N*=1000 to 5000 and are listed by column.

a) Stochastic search, E=1.24, H=1.19

Model/N	1000	2000	3000	4000	5000	6000	7000
E+H	4.74E-04	0.047	0.109	0.322	0.473	0.726	0.830
G	0.131	0.310	0.494	0.442	0.392	0.204	0.128
H	0.056	0.139	0.116	0.143	0.085	0.044	9.04E-03
null	0.790	0.479	0.235	0.057	0.014	2.27E-03	0
other	0.023	0.026	0.046	0.036	0.037	0.024	0.033

b) Stepwise regression, E=1.24, H=1.19

Model/N	1000	2000	3000	4000	5000	6000	7000
E	0	0	0	0.07	0.08	0	0.1
G	0	0.08	0.33	0.61	0.68	0.71	0.76
H	0	0	0	0	0.13	0.21	0.14
null	1	0.91	0.64	0.27	0.11	0	0
other	0	0.01	0.03	0.05	0	0.08	0

c) Stochastic search, E=1.19, H=1.24

Model/N	1000	2000	3000	4000	5000	6000	7000
E+H	2.18E-03	7.94E-03	0.063	0.108	0.201	0.385	0.506
G	0.170	0.316	0.309	0.460	0.328	0.221	0.214
H	0.159	0.404	0.571	0.416	0.450	0.372	0.253
null	0.654	0.246	0.041	3.03E-03	0	0	0
other	0.015	0.026	0.016	0.013	0.020	0.022	0.027

d) Stepwise regression, E=1.19, H=1.24

Model/N	1000	2000	3000	4000	5000	6000	7000
G	0.03	0.11	0.28	0.53	0.46	0.35	0.38
H	0	0.08	0.48	0.44	0.54	0.65	0.62
null	0.97	0.81	0.24	0	0	0	0
other	0	0	0	0.03	0	0	0

Supplementary Table 6: Case-control simulations with two causal variants, E+H, in *CTLA4*. **(a,c)** Model mean posterior probability (GUESSFM; stochastic search) and **(b,d)** Mean model selection probability (stepwise regression) for simulated data having causal variants E + H with odds ratios E:1.24, H:1.19 **(a,b)** and E:1.19, H:1.24 **(c,d).** Case-control data were simulated with the characteristics of the *CTLA4* region, and there were 100 replications. Sample sizes were *N* cases, *N* controls for *N*=1000 to 7000 and are listed by column.

a) Stochastic search, B=0.8

Model/N	1000	2000	3000	4000	5000
A	0.011	0.015	0.024	3.66E-03	1.39E-03
B	0.047	0.420	0.680	0.875	0.979
D	0.029	0.056	0.050	0.03351238	0.012
A+B	2.56E-05	2.95E-04	6.91E-04	5.72E-04	7.87E-04
A+D	2.36E-05	6.22E-04	9.27E-04	3.07E-03	5.45E-04
B+D	3.14E-05	3.21E-04	6.02E-04	2.98E-04	1.76E-03
A+B+D	0	0	0	0	0
null	0.903	0.486	0.235	0.068	2.74E-03
other	9.64E-03	0.022	8.83E-03	0.017	2.16E-03

b) Stepwise regression, B=0.8

Model/N	1000	2000	3000	4000	5000
A	0	0	0	0	0
B	0.01	0.23	0.48	0.88	1
D	0	0.01	0.03	0.02	0
A+B	0	0	0	0	0
A+D	0	0	0	0	0
B+D	0	0	0	0	0
A+B+D	0	0	0	0	0
null	0.99	0.76	0.49	0.1	0
other	0	0	0	0	0

Supplementary Table 7: Case-control simulations with a single causal variant B in *IL2RA*. **(a)** Model mean posterior probability (GUESSFM; stochastic search) and **(b)** Mean model selection probability (stepwise regression) for simulated data having causal variant B with OR=0.8. Data were simulated with the characteristics of the *IL2RA* region and there were 100 replications. Sample sizes were *N* cases, *N* controls for *N*=1000 to 5000 and are listed by column.

a) Stochastic search, A=0.84, D=0.77

Model\N	1000	2000	3000	4000	5000	6000	7000
null	0.622	0.168	0.110	0.062	0.072	0.031	0.024
A	6.43E-04	2.90E-03	7.50E-03	0.017	0.020	0.043	0.054
A+D	2.87E-03	0.043	0.072	0.178	0.265	0.422	0.492
B	0.103	0.209	0.183	0.197	0.141	0.174	0.205
D	0.243	0.537	0.579	0.472	0.476	0.267	0.160
other	0.028	0.041	0.049	0.074	0.026	0.062	0.065

b) Stepwise regression, A=0.84, D=0.77

Model\N	1000	2000	3000	4000	5000	6000	7000
null	1	0.700	0.360	0.150	0.120	0.080	0.020
B	0	0.130	0.190	0.370	0.280	0.380	0.390
D	0	0.170	0.440	0.480	0.600	0.530	0.570
other	0	0	0.010	0	0	0.010	0.020

c) Stochastic search, A=0.81, D=0.8

Model\N	1000	2000	3000	4000	5000	6000	7000
null	0.542	0.317	0.090	0.041	0.037	0.024	0.009
A	0.011	8.44E-03	0.029	0.018	0.025	0.057	0.063
A+D	6.64E-03	0.078	0.127	0.226	0.243	0.442	0.523
B	0.159	0.313	0.449	0.503	0.421	0.315	0.327
D	0.158	0.220	0.227	0.195	0.220	0.154	0.057
other	0.123	0.064	0.079	0.017	0.054	8.94E-03	0.022

d) Stepwise regression, A=0.81, D=0.8

Model\N	1000	2000	3000	4000	5000	6000	7000
null	1	0.95	0.61	0.2	0.1	0.1	0.06
B	0	0.04	0.3	0.65	0.62	0.62	0.74
D	0	0	0.06	0.15	0.27	0.28	0.19
other	0	0.01	0.03	0	0.01	0	0.01

e) Stochastic search, A=0.77, D=0.84

Model\N	1000	2000	3000	4000	5000	6000	7000
null	0.849	0.330	0.135	0.021	0.014	6.30E-04	3.54E-03
A	0.034	0.138	0.075	0.103	0.067	0.080	0.049
A+D	1.08E-03	0.031	0.204	0.326	0.342	0.348	0.522
B	0.074	0.403	0.501	0.509	0.540	0.543	0.370
D	8.25E-03	0.051	0.043	0.017	0.011	7.31E-03	0.027
other	0.034	0.047	0.043	0.024	0.025	0.021	0.028

f) Stepwise regression, A=0.77, D=0.84

Model\N	1000	2000	3000	4000	5000	6000	7000
null	1	0.97	0.77	0.46	0.2	0.1	0.01
B	0	0.03	0.19	0.46	0.74	0.82	0.92
other	0	0	0.04	0.08	0.06	0.08	0.07

Supplementary Table 8: Case-control simulations with two causal variants, A+D, in *IL2RA*. **(a,c,e)** Model mean posterior probability (GUESSFM; stochastic search) and **(b,d,f)** Mean model selection probability (stepwise regression) for simulated data having causal variants A and D with odds ratios A:0.84, D:0.77 **(a,b)**, A:0.81, D:0.8 **(c,d)** and A:0.77, D:0.84 **(e,f)**. Data were simulated with the characteristics of the *IL2RA* region and there were 100 replications. Sample sizes were *N* cases, *N* controls for *N*=1000 to 7000 and are listed by column.
