# Supplementary material for: Stochastic search and joint fine-mapping increases accuracy and identifies previously unreported associations in immune-mediated diseases
Source: Nat Commun. 2019 Jul 19;10:3216. doi: 10.1038/s41467-019-11271-0 (PMC6642100; doi:10.1038/s41467-019-11271-0)
Supplement: Supplementary file 14 — Reporting Summary [file 41467_2019_11271_MOESM14_ESM.pdf]

nature research

Corresponding author(s): Jennifer Asimit, Chris Wallace

Last updated by author(s): 25/6/19

Reporting Summary

Nature Research wishes to improve the reproducibility of the work that we publish. This form provides structure for consistency and transparency in reporting. For further information on Nature Research policies, see Authors & Referees and the Editorial Policy Checklist.

Statistics

For all statistical analyses, confirm that the following items are present in the figure legend, table legend, main text, or Methods section.

n/a	Confirmed	
☐	☒	The exact sample size (*n*) for each experimental group/condition, given as a discrete number and unit of measurement
☒	☐	A statement on whether measurements were taken from distinct samples or whether the same sample was measured repeatedly
☐	☒	The statistical test(s) used AND whether they are one- or two-sided<br>*Only common tests should be described solely by name; describe more complex techniques in the Methods section.*
☐	☒	A description of all covariates tested
☐	☒	A description of any assumptions or corrections, such as tests of normality and adjustment for multiple comparisons
☐	☒	A full description of the statistical parameters including central tendency (e.g. means) or other basic estimates (e.g. regression coefficient) AND variation (e.g. standard deviation) or associated estimates of uncertainty (e.g. confidence intervals)
☒	☐	For null hypothesis testing, the test statistic (e.g. *F*, *t*, *r*) with confidence intervals, effect sizes, degrees of freedom and *P* value noted<br>*Give P values as exact values whenever suitable.*
☐	☒	For Bayesian analysis, information on the choice of priors and Markov chain Monte Carlo settings
☒	☐	For hierarchical and complex designs, identification of the appropriate level for tests and full reporting of outcomes
☒	☐	Estimates of effect sizes (e.g. Cohen's *d*, Pearson's *r*), indicating how they were calculated

Our web collection on statistics for biologists contains articles on many of the points above.

Software and code

Policy information about availability of computer code

Data collection	No software was used for data collection.
Data analysis	Our proposed method MFM is available for use at https://jennasimit.github.io/MFM/ and simulation code is available at https://jennasimit.github.io/MFMextra/. Custom code for our analyses is available at https://github.com/chr1swallace/MFM-analysis and https://github.com/chr1swallace/MFM-paper.

For manuscripts utilizing custom algorithms or software that are central to the research but not yet described in published literature, software must be made available to editors/reviewers. We strongly encourage code deposition in a community repository (e.g. GitHub). See the Nature Research guidelines for submitting code & software for further information.

Data

Policy information about availability of data

All manuscripts must include a data availability statement. This statement should provide the following information, where applicable:

- Accession codes, unique identifiers, or web links for publicly available datasets
- A list of figures that have associated raw data
- A description of any restrictions on data availability

Complete results from our analyses have been deposited at figshare under DOI 10.6084/m9.figshare.8289677 [https://figshare.com/articles/MFM-output/8289677] and are also available at https://chr1swallace.github.io/MFM-output/index.html. Data was obtained from the study authors for each of the six autoimmune diseases that we analysed. There are no restrictions for data access and the following may be requested from the original study authors: ATD ImmunoChip, Cooper et al. (https://www.ncbi.nlm.nih.gov/pubmed/22922229); RA ImmunoChip, Eyre et al. (https://www.ncbi.nlm.nih.gov/pmc/articles/PMC3882906); JIA ImmunoChip, Hinks et al. (https://www.ncbi.nlm.nih.gov/pubmed/23603761). MS ImmunoChip data was accessed through application to the International Multiple Sclerosis Genetic Consortium (IMSGC; http://www.imsgenetics.org/). Primary analysis of the MS data is presented by IMSGC (https://www.ncbi.nlm.nih.gov/pmc/articles/PMC3832895/). The primary analysis of the Celiac ImmunoChip is by Trynka et al. (https://www.nature.com/articles/ng.998) and the genotype data is hosted by the European Bioinformatics Institute, under accession number EGAS00000000053 [https://www.ebi.ac.uk/ega/studies/EGAS00000000053] . T1D ImmunoChip data is

available from dbGaP (Study Accession: phs000180.v3.p2 [https://www.ncbi.nlm.nih.gov/projects/gap/cgi-bin/study.cgi?study_id=phs000180.v3.p2]) and 2000 T1D samples were genotyped as part of the WTCCC (and controls) - data access is described at https://www.wtccc.org.uk/info/access_to_data_samples.html.

Field-specific reporting

Please select the one below that is the best fit for your research. If you are not sure, read the appropriate sections before making your selection.

☒ Life sciences ☐ Behavioural & social sciences ☐ Ecological, evolutionary & environmental sciences

For a reference copy of the document with all sections, see nature.com/documents/nr-reporting-summary-flat.pdf

Life sciences study design

All studies must disclose on these points even when the disclosure is negative.

Sample size	We used already collected samples, which were deemed powerful enough for individual studies.
Data exclusions	To ensure controls could be combined across datasets, we restricted analysis for the multinomial model to UK samples, and used principal component analysis including 1000 Genomes data to exclude 2 individuals who fell outside individual country clusters.
Replication	There was no replication, as there is no gold standard for replication of fine-mapping results. We identify likely causal variants and provide posterior probabilities of support for groups of genetic variants.
Randomization	This was not relevant to our study as individuals were assigned to groups according to disease status.
Blinding	Blinding was not relevant for the same reasons as above.

Reporting for specific materials, systems and methods

We require information from authors about some types of materials, experimental systems and methods used in many studies. Here, indicate whether each material, system or method listed is relevant to your study. If you are not sure if a list item applies to your research, read the appropriate section before selecting a response.

Materials & experimental systems

n/a	Involved in the study	
☒	☐	Antibodies
☒	☐	Eukaryotic cell lines
☒	☐	Palaeontology
☒	☐	Animals and other organisms
☒	☐	Human research participants
☒	☐	Clinical data

Methods

n/a	Involved in the study	
☒	☐	ChIP-seq
☒	☐	Flow cytometry
☒	☐	MRI-based neuroimaging
