# Supplementary material for: Stochastic search and joint fine-mapping increases accuracy and identifies previously unreported associations in immune-mediated diseases
Source: Nat Commun. 2019 Jul 19;10:3216. doi: 10.1038/s41467-019-11271-0 (PMC6642100; doi:10.1038/s41467-019-11271-0)
Supplement: Supplementary file 15 — Description of Additional Supplementary Files [file 41467_2019_11271_MOESM15_ESM.docx]

**Title:** **Supplementary Data 1: Selection of region-disease combinations for fine-mapping.**
**Description:** Each of the 90 regions is listed together with chromosome, start and end positions and minimum p-values for SNP association with each of the six autoimmune diseases (UK) and for RA and CEL in international samples (iCEL, iRA). Region-disease combinations with minimum p-values < 10E-6 were selected for fine-mapping and are in bold font.

**Title:** **Supplementary Data 2: Stepwise results for all regions and autoimmune diseases that met criteria for fine-mapping.
Description:** For each region and disease, the SNPs selected by stepwise search are listed, as well as their conditional p value and order of selection.

**Title:** **Supplementary Data 3: Stochastic search results for all regions that met criteria for fine-mapping**.
**Description:** For each region, SNPs from each SNP group are listed, together with base pairs position (GRCh37/hg19 assembly), alleles, MAF in UK samples, and marginal posterior probability of inclusion. At the first SNP in each group, the marginal group posterior probability of inclusion is listed in the Total column.

**Title:** **Supplementary Data 4: Potential frequency of joint tagging.
Description:** For each region analysed in this study (reg), this table shows the number of SNPs (nsnps) with MAF > thr (always 0.05), the number of potential tag pairs considered (maxtags), the number of these for which at least one additional SNP exists which they may tag according to Supp Note 1, eqn 3 (tags) or eqn 4 (tags.f). maxpatt shows the number of potential SNP trios considered (two tags + 1 SNP to be tagged), the number of these which correspond to a tagging pattern according to Supp Note 1, eqn 3(patt) or eqn 4 (patt.f). This data is presented in Figure 2.

**Title:** **Supplementary Data 5: Simulations of two diseases with one strong effect shared causal variant.
Description:** Disease 1 was simulated to have causal variants A + D (odds ratios A:1.4,D:1.25) and disease 2 had causal variants A + C (odds ratios A:1.4, C:1.25). MFM was run at a range of target odds (TO; prior odds of no sharing of causal variants between one disease and any other disease) values to illustrate the impact of TO and with decreasing TO there is an increasing prior weight for sharing of variants; TO=null indicates no sharing and independent stochastic search analyses were run and TO=1 was the setting used in our MFM analyses.

**Title:** **Supplementary Data 6: Simulations of two diseases with one weak effect shared causal variant.**
**Description:** Disease 1 was simulated to have causal variants A and D (odds ratios A:1.25,D:1.4) and disease 2 had causal variants A + C (odds ratios A:1.25, C:1.4). MFM is run at a range of target odds (TO; prior odds of no sharing of causal variants between one disease and any other disease) values to illustrate the impact of TO and with decreasing TO there is an increasing prior weight for sharing of variants; TO=null indicates no sharing and independent stochastic search analyses were run and TO=1 was the setting used in our MFM analyses.

**Title:** **Supplementary Data 7:Simulations of two diseases with distinct causal variants.** **Description:** Disease 1 was simulated to have causal variants A + D (A:1.25, D:1.25) and disease 2 had single causal variant C (OR 1.25). MFM is run at a range of target odds (TO; prior odds of no sharing of causal variants between one disease and any other disease) values to illustrate the impact of TO and with decreasing TO there is an increasing prior weight for sharing of variants; TO=null indicates no sharing and independent stochastic search analyses were run and TO=1 was the setting used in our MFM analyses.

**Title:** **Supplementary Data 8: Simulations of two diseases where one disease had no associations.**
**Description:** Disease 1 was simulated to have causal variants A and D (odds ratios A:1.4, D:1.4) and disease 2 had no causal variants. MFM is run at a range of target odds (TO; prior odds of no sharing of causal variants between one disease and any other disease) values to illustrate the impact of TO and with decreasing TO there is an increasing prior weight for sharing of variants; TO=null indicates no sharing and independent stochastic search analyses were run and TO=1 was the setting used in our MFM analyses.

**Title:** **Supplementary Data 9: MFM results for all regions that met criteria for fine-mapping.
Description:** For each region, SNPs from each SNP group are listed, together with base pairs position (GRCh37/hg19 assembly), alleles, MAF in UK samples, and marginal posterior probability of inclusion from stochastic search, MFM, and MFM with international samples. At the first SNP in each group, the marginal group posterior probability of inclusion is listed in the Total column for each of these analyses.

**Title:** **Supplementary Data 10: Comparison of the BICs for *CTLA4* SNP models. Description:** Representative SNP models from several group models are fit to ATD, CEL, T1D, international-RA and international-CEL and the BIC of the best fitting model for each disease is in bold. The posterior probability (PP) and group PP (GPP) are given for each SNP and SNP group model, with the highest GPP model in bold.

**Title:** **Supplementary Data 11: Details of IL2RA stochastic search and MFM results and the resulting SNP groups.**
**Description:** SNPs within each SNP group are listed, together with their base pairs position (GRCh37/hg19 assembly), alleles and MAF (based on UK controls data). Previous SNP groups (Wallace et al. 2015) are nested within our new SNP groups and these matches are marked. For both stochastic search (SS) and MFM, the marginal posterior probability of inclusion (MPPi) is given for each SNP and disease and the SNP group marginal posterior probability (Total MPPi). For MFM, ATD, MS and T1D analyses are based on UK samples, as well as the inclusion of international controls (int.ATD, int.MS, int.T1D); international RA (iRA) analyses are included, but not RA (UK only) since it did not meet our fine-mapping criteria.

**Title:** **Supplementary Data 12: Allele-specific expression (ASE) analysis results.**
**Description:** The genotype of a SNP from each of the *IL2RA* SNP groups defined in Supplementary Data 11 is listed for each participant that ASE was performed on. The genotypes are phased so that all the SNPs listed for allele 1 are on the same chromosome, and gives directionality for the ASE readout SNP rs12244380, which is in the 3’UTR of IL2RA. ASE was measured using targeted NGS and the counts from each allele of rs12244380 are provided with 3-4 technical replicates performed. The average of the technical replicates was used to calculate the allelic ratio. Some samples were tested multiple times and these are highlighted in green. The allelic ratio for the central memory CD4^+^ T cells and naive CD4^+^ T cells are calculated as the ratio of A to G alleles at the readout SNP, and then re-ordered based on phased haplotypes to match the direction shown as top:bottom in the cartoon haplotypes depicted Figure 7a. Not all samples were tested with both naive and central memory CD4+ T cells due to cell number availability. The genomic DNA samples are included as a control showing there is no bias regardless of genotype and all are reported as the ratio of the A:G allele of rs12244380.
